# Casein Protein Processing Strongly Modulates Post-Prandial Plasma Amino Acid Responses In Vivo in Humans

**DOI:** 10.3390/nu12082299

**Published:** 2020-07-31

**Authors:** Jorn Trommelen, Michelle E. G. Weijzen, Janneau van Kranenburg, Renate A. Ganzevles, Milou Beelen, Lex B. Verdijk, Luc J. C. van Loon

**Affiliations:** 1 NUTRIM School of Nutrition and Translation Research in Metabolism, Maastricht University Medical Centre, P.O. Box 616, 6200 MD Maastricht, The Netherlands; jorn.trommelen@maastrichtuniversity.nl (J.T.); m.weijzen@maastrichtuniversity.nl (M.E.G.W.); j.vankranenburg@maastrichtuniversity.nl (J.v.K.); milou.beelen@maastrichtuniversity.nl (M.B.); Lex.verdijk@maastrichtuniversity.nl (L.B.V.); 2 Top Institute Food and Nutrition (TIFN), 6709 PA Wageningen, The Netherlands; 3 FrieslandCampina, Stationsplein 4, 3818 LE Amstersfoort, The Netherlands; Renate.Ganzevles@frieslandcampina.com

**Keywords:** digestibility, dairy, supplementation, milk, protein synthesis, metabolism, solubility, leucine, coagulation, protein quality

## Abstract

Micellar casein is characterized as a slowly digestible protein source, and its structure can be modulated by various food processing techniques to modify its functional properties. However, little is known about the impact of such modifications on casein protein digestion and amino acid absorption kinetics and the subsequent post-prandial plasma amino acid responses. In the present study, we determined post-prandial aminoacidemia following ingestion of isonitrogenous amounts of casein protein (40 g) provided as micellar casein (Mi-CAS), calcium caseinate (Ca-CAS), or cross-linked sodium caseinate (XL-CAS). Fifteen healthy, young men (age: 26 ± 4 years, BMI: 23 ± 1 kg·m^−2^) participated in this randomized cross-over study and ingested 40 g Mi-Cas, Ca-CAS, and XL-CAS protein, with a ~1 week washout between treatments. On each trial day, arterialized blood samples were collected at regular intervals during a 6 h post-prandial period to assess plasma amino acid concentrations using ultra-performance liquid chromatography. Plasma amino acid concentrations were higher following the ingestion of XL-CAS when compared to Mi-CAS and Ca-CAS from t = 15 to 90 min (all *p* < 0.05). Plasma amino acid concentrations were higher following ingestion of Mi-CAS compared to Ca-CAS from t = 30 to 45 min (both *p* < 0.05). Plasma total amino acids iAUC were higher following the ingestion of XL-CAS when compared to Ca-CAS (294 ± 63 vs. 260 ± 75 mmol·L^−1^, *p* = 0.006), with intermediate values following Mi-CAS ingestion (270 ± 63 mmol·L^−1^, *p* > 0.05). In conclusion, cross-linked sodium caseinate is more rapidly digested when compared to micellar casein and calcium caseinate. Protein processing can strongly modulate the post-prandial rise in plasma amino acid bioavailability in vivo in humans.

## 1. Introduction

It has been well-established that muscle protein turnover is highly responsive to nutrient intake in healthy adults. Protein ingestion increases muscle protein synthesis rates [1,2,3]. The muscle protein synthetic response to protein feeding is regulated on various levels, ranging from protein digestion and amino acid absorption [4,5], the post-prandial rise in insulin and subsequent increase in microvascular recruitment [6,7], amino acid uptake in skeletal muscle [2,8], intramuscular anabolic signaling [9], and the incorporation of dietary protein-derived amino acids into muscle protein [10,11]. The post-prandial rise in muscle protein synthesis can be modulated by various dietary factors, including the type, amount, and timing of protein consumed [12,13].

Milk contains both whey and micellar casein protein (~20 and 80% of total protein content, respectively). Whey protein is characterized as a rapidly digestible protein source, with ingestion resulting in a fast, but transient post-prandial increase in plasma amino acid concentrations [14,15,16]. In contrast, micellar casein is a more slowly digestible protein source with ingestion resulting in a moderate, but more prolonged post-prandial increase in plasma amino acid concentrations [14,15,16]. Whey protein is typically considered more potent at stimulating muscle protein synthesis rates when compared to other types of protein, such as micellar casein [15,17]. This has been, at least partly, attributed to the more rapid protein digestion and amino acid absorption rates following ingestion of whey when compared to micellar casein protein [4,5]. Consequently, it is now generally believed that the digestion and amino acid absorption kinetics of a protein strongly modulates the post-prandial muscle protein synthetic response to feeding.

Casein protein exists in milk as large colloidal particles called casein micelles. Casein micelles coagulate in the stomach due to both the acidic environment and the presence of pepsin [18]. However, the micellar casein structure can also be intentionally disrupted by various food processing techniques to modify its functional properties [19]. Treatment of micellar casein with rennet causes the casein particles to aggregate and form a dense gel (i.e., cheese). Acidification of micellar casein results in a more open gel (i.e., yoghurt). Acidified casein can be further treated by the addition of sodium, potassium, or calcium hydroxide to produce caseinates (sodium caseinate, potassium caseinate, and calcium caseinate, respectively). Caseinates are more soluble compared to rennet casein, acidified casein, or micellar casein [20]. In addition, coagulation behavior of caseinates and micellar casein differ, as caseinate is coagulated only by acidic pH and not by pepsin [19]. The latter explains why caseinate feeding results in faster gastric emptying when compared to micellar casein feeding in vivo in rats [21]. As a consequence, it is generally believed that caseinates are more rapidly digested when compared to micellar casein [22]. We hypothesized that caseinate ingestion leads to a more rapid post-prandial rise in amino acid concentrations when compared to ingestion of micellar casein.

Casein proteins can be further modified by the addition of cross-linking enzymes, such as transglutaminase, to change functional properties, such as stability, firmness, and viscosity [23]. Little is known about whether cross-linking caseinate impacts protein digestion and amino acid absorption rates. Juvonen et al. observed no differences in post-prandial aminoacidemia following the ingestion of cross-linked caseinate when compared to regular caseinate [24]. However, the test beverages contained more carbohydrates than protein, which likely delayed protein digestion and amino acid absorption kinetics [25], thereby masking potential differences in post-prandial aminoacidemia. We hypothesized that ingestion of cross-linked sodium caseinate delays protein digestion and amino acid absorption, resulting in an attenuated post-prandial rise in plasma amino acids when compared to caseinate ingestion.

To assess the impact of protein processing on post-prandial plasma amino acid availability, 15 healthy, young adults were recruited to participate in a study in which we compared the in vivo post-prandial plasma amino acid responses following the ingestion of 40 g micellar casein, calcium caseinate, and cross-linked sodium caseinate.

## 2. Materials and Methods

### 2.1. Subjects

Fifteen healthy, young male subjects were recruited to participate in this randomized cross-over study (age 26 ± 4 years, body weight 77.5 ± 6.1 kg, body mass index: 23 ± 1 kg∙m^−2^). Potential subjects were included if they were non-smoking, recreationally active (exercised ≤3 times per week), and had no history of lactose intolerance or milk protein allergy. The participants were informed about the experimental procedures and possible risks of participation prior to signing an informed consent form. The study was approved by the Medical Ethical Committee of the Maastricht University Medical Centre, Maastricht, The Netherlands (METC153015), and was registered at the Netherlands Trial Register (NTR5242). All procedures were carried out in accordance with the standards stated in the 2013 version of the Helsinki Declaration. The study was independently monitored by the Clinical Trial Center Maastricht.

### 2.2. Design

This double-blind, randomized, cross-over study assessed the post-prandial plasma amino acid responses to the ingestion to three different types of casein protein: micellar casein, calcium caseinate, and cross-linked sodium caseinate. Subjects performed three tests separated by at least one week. During each test, subjects ingested one of three different casein protein powders dissolved in water and were studied during a 6 h assessment period throughout which blood samples were collected while the subjects remained in a seated position.

### 2.3. Pretesting

Prior to inclusion in the study, subjects first completed a screening session which consisted of assessment of body weight and height, as well as body composition by dual-energy X-ray absorptiometry (DEXA) scan (Discovery A, Hologic, Bedford, MA, USA). All subjects were deemed healthy based on their response to a routine medical screening questionnaire.

### 2.4. Standardization of Physical Activity and Diet

Participants refrained from strenuous physical activity in the 48 h leading up to the trial days. Physical activity and dietary intake were recorded during 48 h prior to the first experimental test day, and were replicated in the 48 h prior to the second and third test day. On the evening prior to each of the three test days, all participants consumed a standardized packaged dinner (54 kJ/kg) that was based on body weight, providing ~60% energy as carbohydrate, ~26% energy as fat, and ~14% energy as protein.

### 2.5. Experimental Procedures

**Figure 1:**
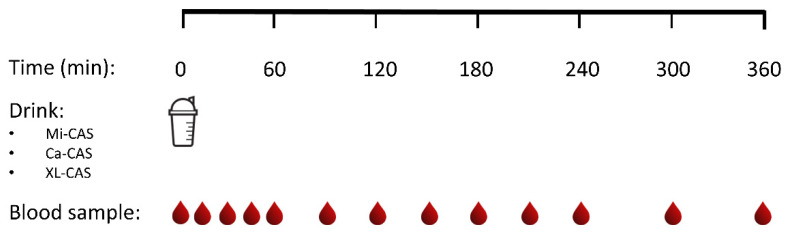
Schematic study representation. Ca-CAS: caseinate protein; XL-CAS: cross-linked casein protein; Mi-CAS: micellar casein protein.

Subjects reported to the laboratory by car or public transport at 8:00 a.m. on trial days following an overnight fast. The experimental protocol during each test day is shown in Figure 1. Each test day started by placement of a Teflon catheter into a dorsal hand vein for arterialized blood draws. To allow sampling of arterialized blood, the hand was first placed in a hot box (60 °C) for 10 min before drawing blood. After collection of a basal blood sample, participants consumed one of the casein protein powders within a 5 min period (t = 0 min). Consumption of the casein protein powders was then followed by a 6 h post-prandial period in which 12 blood samples were collected at t = 15, 30, 45, 60, 90, 120, 150, 180, 210, 240, 300, and 360 min while the subjects remained in an upright position [26,27].

### 2.6. Protein

The protein ingredients assessed in this study were specifically produced for research purposes within the Top Institute Food and Nutrition (TIFN) public–private partnership. The protein powders were micellar casein (89.9% protein: NIZO food research B.V., Ede, The Netherlands), calcium caseinate (90.5% protein: FrieslandCampina Innovation Centre, Wageningen, The Netherlands) and cross-linked sodium caseinate (91.1% protein: FrieslandCampina Innovation Centre, Wageningen, The Netherlands). Each protein powder bolus provided 40 g protein (total nitrogen content × 6.38) dissolved in water to a total volume of 600 mL. The final test beverages were flavored by adding 3 mL vanilla flavour (Dr Oetker, Amersfoort, The Netherlands).

### 2.7. Plasma Analysis

Arterialized blood samples (8 mL) were collected in EDTA-containing tubes and immediately centrifuged at 1000× *g* for 10 min at 4 °C. Aliquots of plasma were frozen in liquid nitrogen and stored at −80 °C for subsequent analyses. Plasma glucose and insulin concentrations were analyzed using commercially available kits (GLUC3, cat. no. 05168791190, intra- and inter-assay variation: 1.2 and 1.2%, respectively; and Immunologic, cat. no. 12017547122; Roche, intra- and inter-assay variation: 3.2 and 4.2%, respectively). Quantification of plasma amino acids was performed using ultra-performance liquid chromatograph mass spectrometry (UPLC-MS; ACQUITY UPLC H-Class with QDa; Waters, Saint-Quentin, France). Fifty µL of plasma was deproteinized using 100 µL of 10% SSA with 50 µM of MSK-A2 internal standard (Cambridge Isotope Laboratories, Massachusetts, USA). Subsequently, 50 µL of ultra-pure demineralized water was added, and samples were centrifuged. After centrifugation, 10 µL of supernatant was added to 70 µL of Borate reaction buffer (Waters, Saint-Quentin, France). In addition, 20 µL of AccQ-Tag derivatizing reagent solution (Waters, Saint-Quentin, France) was added after which the solution was heated to 55 °C for 10 min. Of this 100 µL derivative, 1 µL was injected and measured using UPLC-MS.

### 2.8. Solubility of Protein Ingredients

Post-hoc solubility analyses were performed on the casein test products. Protein solutions (500 g, 6% *w*/*w* protein) were prepared by dispersing protein powder into demineralized water of 50 °C with Mars stirrer for 5 min at 350 rpm. The dispersions were transferred into 500 mL Scott bottles for hydration and cooling down to room temperature, while being stirred, for 2 h. Duplicate subsamples of 45 g were weighed and centrifuged for 15 min at 2900× *g* using a Varifuge. After pouring of the supernatants and weighing, the wet sediments were dried over a weekend in an oven at 90 °C and weighed again. With the assumption that the mass of the dried pellet is purely protein, the following formula expresses the protein solubility (σ) in a percentage:

σ = (TPM − MP)/TPM × 100%,(1)

where TPM is the total protein mass in the subsample and MP is the weight of the dry pellet. Solubility was 99%, 53%, and 5% for cross-linked sodium caseinate, calcium caseinate, and micellar casein protein, respectively.

### 2.9. Statistical Analysis

All data are expressed as means ± SD. Plasma glucose, insulin, and amino acid concentrations were analyzed by a two-factor repeated-measures ANOVA with time and treatment as within-subject factors. Incremental area under the curves (calculated by the linear trapezoidal method), maximal concentration, and time to maximal concentrations were analyzed using a one-way repeated measures ANOVA with treatment as a within-subject factor. The observed main effect or interactions were further assessed with Bonferroni-corrected testing where appropriate. Statistical significance was set at *p* < 0.05. All calculations were performed using SPSS (version 25.0, IBM, Armonk, NY, USA).

## 3. Results

### 3.1. Plasma Glucose and Insulin Concentrations

**Figure 2:**
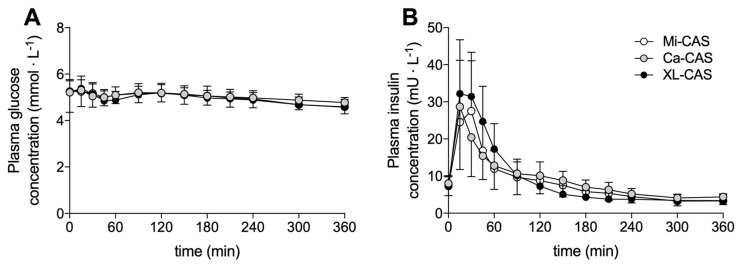
Plasma glucose (**A**) and insulin (**B**) concentrations. Means ± SD (*n* = 15). Ca-CAS: caseinate protein; XL-CAS: cross-linked casein protein; Mi-CAS: micellar casein protein.

Plasma glucose and plasma insulin concentrations are shown in Figure 2. Plasma glucose concentrations at t = 0 min averaged 5.2 ± 0.9, 5.2 ± 0.5, and 5.2 ± 0.5 mmol∙L^−1^ in the calcium caseinate, cross-linked sodium caseinate, and micellar casein treatment, respectively. Plasma glucose concentrations decreased slightly following protein ingestion (main effect time: *p* < 0.001), but no significant differences were observed between the treatments (time x treatment interaction: *p* = 0.079, treatment effect: *p* = 0.327). Plasma insulin concentrations at t = 0 min averaged 8.0 ± 3.3, 7.2 ± 2.7, and 7.3 ± 0.7 mU∙L^−1^ in the calcium caseinate, cross-linked sodium caseinate, and the micellar casein treatment, respectively. Plasma insulin concentrations increased rapidly, but transiently, following protein ingestion (main effect time: *p* < 0.001). The increase in plasma insulin was largest following the ingestion of cross-linked sodium caseinate (time x treatment interaction: *p* = 0.002).

### 3.2. Plasma Amino Acid Concentrations

**Figure 3:**
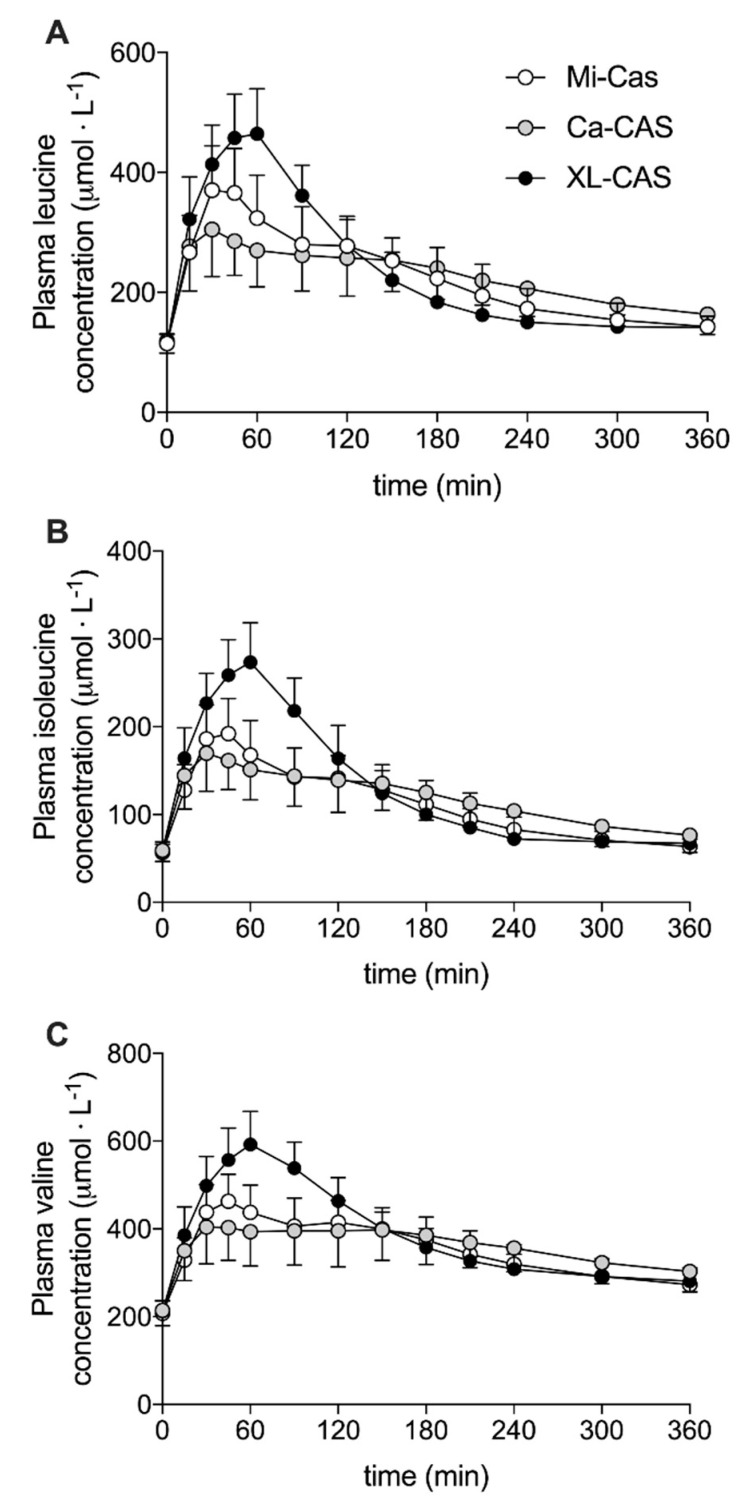
Plasma branched-chain amino acids concentrations: leucine (**A**), isoleucine (**B**), and valine (**C**). Means ± SD (*n* = 15). Ca-CAS: caseinate protein; XL-CAS: cross-linked casein protein; Mi-CAS: micellar casein protein.

**Figure 4:**
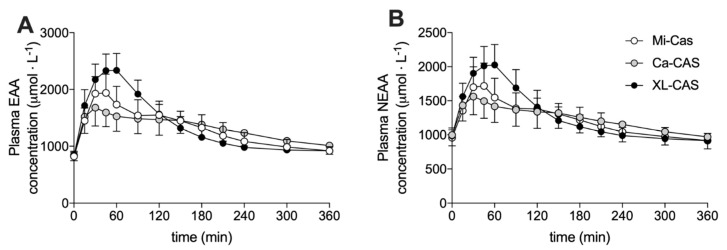
Plasma essential amino acid (**A**) and non-essential amino acid (**B**) concentrations. Means ± SD (*n* = 15). Ca-CAS: caseinate protein; XL-CAS: cross-linked casein protein; Mi-CAS: micellar casein protein.

Plasma concentrations of the branched-chain amino acids (leucine, isoleucine, and valine) are shown in Figure 3. Changes in plasma leucine, isoleucine, and valine concentrations all showed the same general pattern. The ingestion of all casein proteins stimulated a rise in plasma branched-chain amino acid concentrations that peaked ~60 min following ingestion (time effect: *p* < 0.001). The increase in plasma branched-chain amino acid concentrations was most pronounced following cross-linked sodium caseinate ingestion (time x treatment interaction: *p* < 0.001; post-hoc comparisons: cross-linked sodium caseinate > micellar casein and calcium caseinate: all *p* < 0.05) from t = 30 to 90 min. However, this pattern was reversed with slightly higher plasma branch-chain amino acid concentrations in the calcium caseinate treatment compared to the cross-linked sodium caseinate treatment from approximately t = 180 min onwards. Plasma concentrations of essential amino acids (EAA) and non-essential amino acids (NEAA) are shown in Figure 4A,B, respectively. Similar to the plasma branched-chain amino acid response, protein ingestion resulted in a rapid increase in plasma EAA and NEAA that was greatest following XL-CAS ingestion (time x treatment interaction: *p* < 0.001; post-hoc comparisons: cross-linked sodium caseinate > calcium caseinate and micellar casein: *p* < 0.05) from t = 30 to 90 min. Cross-linked sodium caseinate ingestion resulted in a higher plasma EAA iAUC compared to calcium caseinate (+12 ± 17%) and micellar casein (+9 ± 11%) ingestion (*p* < 0.05). In addition, plasma NEAA iAUC was higher following cross-linked sodium caseinate ingestion when compared to calcium caseinate ingestion (*p* = 0.005), but not when compared to micellar casein ingestion (*p* = 0.306). An overview of the post-prandial responses of the individual (non-BCAA) essential and non-essential amino acids are provided in Figures S1 and S2.

## 4. Discussion

The current study assessed the plasma amino acid response to the ingestion of three isonitrogenous powders containing differently processed casein proteins: micellar casein, calcium caseinate, and cross-linked sodium caseinate. The ingestion of all three proteins resulted in a substantial rise in plasma amino acid concentrations; however, the pattern of post-prandial aminoacidemia was vastly different between treatments. The ingestion of cross-linked caseinate resulted in higher peak plasma amino acid concentrations when compared to micellar casein and calcium caseinate (approximately +35 and +20%, respectively). In contrast, plasma amino acid concentrations were ~5–15% lower in the second half of the 6 h post-prandial period following the ingestion of cross-linked sodium caseinate when compared to micellar casein and calcium caseinate.

Micellar casein is classified as a slowly digestible protein source with ingestion resulting in a more moderate, but prolonged post-prandial increase in plasma amino acid concentrations [14,15,16]. Following micellar protein ingestion, we observed a rapid rise in plasma total amino acid concentrations that peaked at 48 ± 38 min, after which plasma amino acid concentrations rapidly declined (Figure 3 and Figure 4; Table 1). Our observations are in line with several other studies from our laboratory [28,29,30], as well as others [31,32,33] that have observed similar plasma amino acid responses following the ingestion of micellar casein. However, in the literature, there have been vast differences reported in the post-prandial plasma amino acid responses following the ingestion of (micellar) casein protein [2,14,16,17,34,35,36,37,38]. There is no clear explanation for this discrepancy in the literature on the post-prandial amino acid responses following the ingestion of various (micellar) casein proteins. However, a possible explanation could be the large variability in solubility reported for milk protein powders (25–100%) [39]. Therefore, we decided to assess the solubility of the investigated powders. In line with our expectations, we observed a very low solubility (5%) for the micellar casein protein assessed in this study. This low solubility may have resulted from specific processing and/or storage conditions [40,41]. It could be speculated that the low solubility of the applied micellar casein may have affected the formation of a coagulum in the stomach, and as such, resulted in more rapid protein digestion and amino acid absorption kinetics. Clearly, more work is needed to identify which factors determine the solubility of milk proteins and whether this impacts their subsequent gastric emptying, protein digestion, and amino acid absorption rate.

A common approach to modulate the protein digestion kinetics of micellar casein is the production of caseinates [20]. In the present study, we assessed the plasma amino acid response following ingestion of a calcium caseinate produced by the addition of calcium hydroxide to acidified micellar casein [20]. We speculated that the ingestion of calcium caseinate would result in more rapid protein digestion and amino acid absorption and, as such, a greater post-prandial plasma amino acid response when compared to micellar casein. In contrast to our hypothesis, we observed a higher post-prandial rise in circulating amino acids following the ingestion of micellar casein when compared to the ingestion of an isonitrogenous amount of calcium caseinate (Figure 3 and Figure 4; Table 1). This can be attributed in part to a more substantial rise in plasma amino acid concentrations following ingestion of micellar casein protein than we anticipated, as micellar casein is considered a typical slowly digestible protein. As previously mentioned, the relatively rapid protein digestion and amino acid absorption of micellar casein in the current study may be attributed to its low solubility (micellar casein 5% and calcium caseinate 53%). In addition, the post-prandial rise in circulating amino acids following ingestion of the caseinate was lower than expected. This may be attributed to the specific salt form of the applied caseinate. Casein(ate) proteins are known to precipitate in the presence of calcium [42] and may cause (more) clotting in the stomach and delayed gastric emptying. Therefore, calcium caseinate may have slower protein digestion and amino acid absorption kinetics when compared to other caseinates. Comparisons between different caseinates should be performed in the future to understand potential differences in their protein digestion and amino acid absorption rates in vivo in humans.

Besides the addition of calcium hydroxide to acidified micellar casein, another approach to modulate the functional properties of micellar casein is enzymatic cross-linking [23]. Transglutaminase can form bonds between glutamine and lysine, thereby altering the protein structure. Micellar casein was processed to generate cross-linked casein, which our subjects ingested. We anticipated that ingestion of the cross-linked casein would delay protein digestion and amino acid absorption and, as such, result in a more blunted post-prandial rise in circulating amino acid concentrations. To our surprise, ingestion of 40 g of cross-linked sodium caseinate resulted in a more rapid rise in circulating plasma amino acid concentrations when compared to the ingestion of calcium caseinate and micellar casein (Figure 3). In line with this, the plasma total amino acids incremental area under the curve (iAUC) was higher following the ingestion of cross-linked sodium caseinate when compared to calcium caseinate (294 ± 63 vs. 260 ± 75 mmol·L^−1^, *p* = 0.006), with intermediate values following micellar casein ingestion (270 ± 63 mmol·L^−1^, *p* > 0.05; Figure 4; Table 1). These data suggest that protein cross-linking may accelerate gastric emptying and/or protein digestion and amino acid absorption rates. So far, only one study has assessed the impact of protein cross-linking on the plasma amino acid response to protein ingestion. Juvonen et al. did not observe differences in the plasma amino responses following ingestion of carbohydrates (40 g) with either cross-linked sodium caseinate (30 g) or regular sodium caseinate (30 g) [24]. These data appear to be in contrast with our present findings. However, carbohydrate co-ingestion has been shown to delay gastric emptying and subsequent protein digestion and amino acid absorption kinetics [25] and, therefore, their study design may have prevented them from detecting differences in post-prandial plasma amino acid responses. However, it is unlikely that carbohydrate co-ingestion would completely mask the large differences in plasma amino acid responses that we observed following the ingestion of cross-linked sodium caseinate and calcium caseinate in the present study. It could be speculated that the observed differences may not be (solely) due the protein cross-linking modification, but may also be modulated by the salt form of caseinate that was used. As mentioned before, calcium caseinate might be more prone to clotting in the stomach due to its high calcium content [42], which would result in a post-prandial amino acid response that reflects a more slowly digestible protein source. Such an explanation would also be consistent with our observation that (cross-linked) sodium caseinate may be more rapidly digested when compared to micellar casein. Clearly, more comparisons between different caseinates should be performed in the future to understand the impact of acidification of micellar casein and the addition of sodium versus calcium hydroxide to modulate protein digestion and amino acid absorption in vivo in humans.

Our data clearly demonstrate that food processing of protein ingredients can strongly modulate the post-prandial amino acid response to protein ingestion, even when micellar casein is used as the original protein source. Various forms of processing, such as heating, separation, added salts, cross-linking, drying, and glycation may all modulate the functional properties of dietary protein [43,44]. Furthermore, some of these processes may even occur during the storage of protein [40,41]. All of these aspects of protein processing and storage, and the lack of reporting on them, may explain the large discrepancies in post-prandial plasma amino acid profiles, seemingly following ingestion of the same protein source. It seems evident that work is needed to address the present processing and storage procedures of protein sources, as well as high-protein foods and their impact on protein digestion and amino acid absorption kinetics and their subsequent post-prandial plasma amino acid availability in vivo in humans.

## 5. Conclusions

The ingestion of cross-linked sodium caseinate results in a more rapid post-prandial rise in circulating plasma amino acid concentrations and a greater plasma amino acid incremental area under the curve when compared to the ingestion of an isonitrogenic amount of calcium caseinate or micellar casein protein. Food processing of protein sources can strongly modulate post-prandial plasma amino acid availability in vivo in humans.

## Figures and Tables

**Table 1:** Plasma glucose, insulin, and amino acid concentrations.

Plasma Metabolites	Micellar Casein			Calcium Caseinate			Cross-Linked Sodium Caseinate		
Glucose									
Cmax (mmol·L^−1^)	5.4	±	0.5 ^a^	5.5	±	0.8 ^a^	5.5	±	0.5 ^a^
Tmax (min)	23	±	31 ^a^	61	±	64 ^a^	28	±	38 ^a^
iAUC (mmol·L^−1^·6 h^−1^)	−92	±	73 ^a^	−67	±	196 ^a^	−89	±	80 ^a^
Insulin									
Cmax (mU·L^−1^)	31.3	±	15.7 ^a^	30.2	±	16.2 ^a^	34.6	±	13.5 ^a^
Tmax (min)	28	±	8 ^a^	28	±	34 ^a^	24	±	8 ^a^
iAUC (mU·L^−1^·6 h^−1^)	321	±	660 ^a^	419	±	653 ^a^	619	±	658 ^a^
Branched-chain amino acids									
Cmax (µmol·L^−1^)	1074	±	148 ^a^	949	±	156 ^a^	1351	±	178 ^b^
Tmax (min)	72	±	58 ^a^	76	±	74 ^a^	57	±	6 ^a^
iAUC (mmol·L^−1^·6 h^−1^)	113	±	23 ^a^	114	±	26 ^a^	132	±	21 ^b^
Essential amino acids									
Cmax (µmol·L^−1^)	2028	±	301 ^c^	1775	±	226 ^a^	2394	±	275 ^b^
Tmax (min)	47	±	38 ^a^	71	±	76 ^a^	56	±	7 ^a^
iAUC (mmol·L^−1^·6 h^−1^)	176	±	38 ^a^	174	±	43 ^a^	190	±	36 ^b^
Non-essential									
Cmax (µmol·L^−1^)	1779	±	316 ^a^	1623	±	203 ^a^	2063	±	287 ^b^
Tmax (min)	48	±	38 ^a^	74	±	76 ^a^	57	±	6 ^a^
iAUC (mmol·L^−1^·6 h^−1^)	94	±	28 ^a,b^	86	±	36 ^a^	104	±	31 ^b^
Total amino acids									
Cmax (µmol·L^−1^)	3804	±	604 ^a^	3394	±	398 ^a^	4454	±	518 ^b^
Tmax (min)	48	±	38 ^a^	69	±	75 ^a^	56	±	7 ^a^
iAUC (mmol·L^−1^·6 h^−1^)	270	±	63 ^a,b^	260	±	75 ^a^	294	±	63 ^b^

Values are mean ± SD, *n* = 15 per treatment. ^a b c^: treatments without a common superscript letter differ, *p* < 0.05. Cmax: maximal concentration. Tmax: time to reach maximal concentration, iAUC: incremental area under the curve.

## Supplementary Materials

The following are available online at https://www.mdpi.com/2072-6643/12/8/2299/s1, Figure S1: Overview of individual (non-BCAA) plasma essential amino acid concentrations. Figure S2: Overview of individual non-plasma non-essential amino acid concentrations.
