# Supplementary material for: Casein Protein Processing Strongly Modulates Post-Prandial Plasma Amino Acid Responses In Vivo in Humans
Source: Nutrients. 2020 Jul 31;12(8):2299. doi: 10.3390/nu12082299 (PMC7468913; doi:10.3390/nu12082299)
Supplement: Supplementary file 1 [file nutrients-12-02299-s001.pdf]

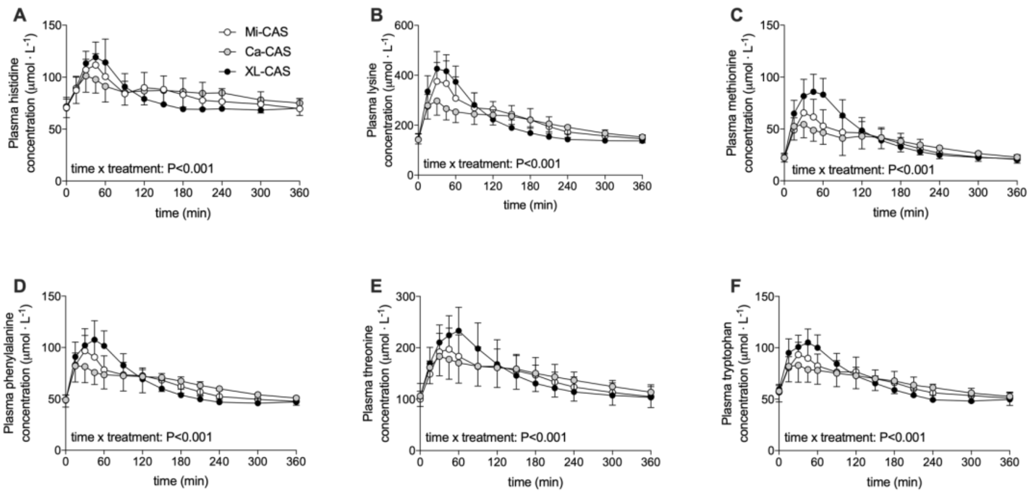

Figure S1. Overview of individual (non-BCAA) plasma essential amino acid concentrations.

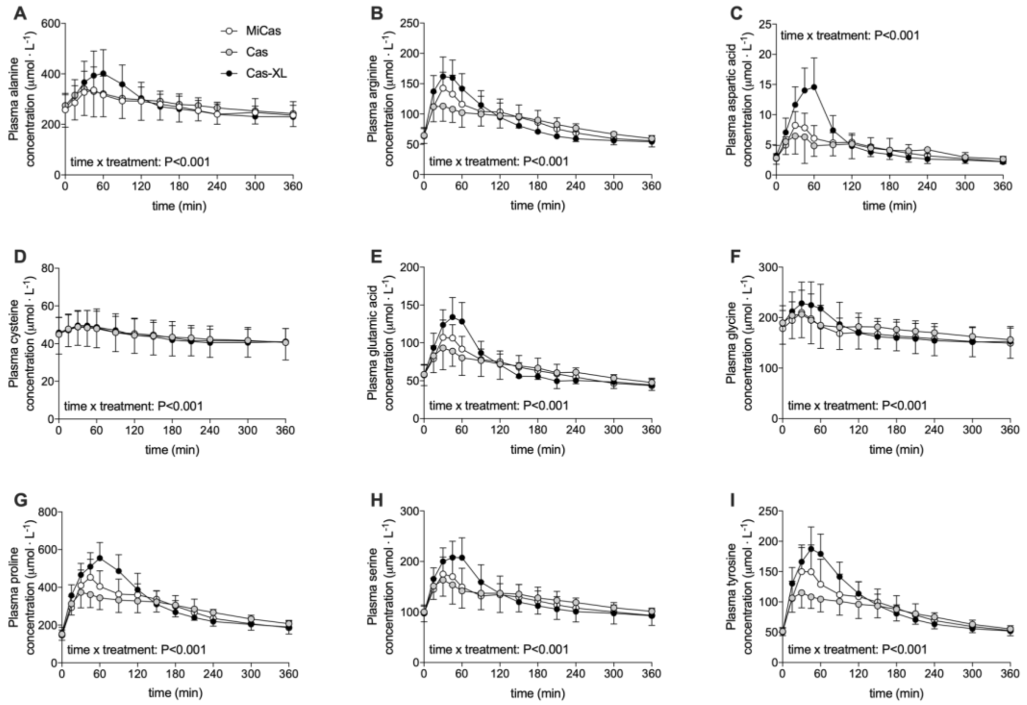

Figure S2. Overview of individual non-plasma non-essential amino acid concentrations.
